# Draw Tower Optical Fibers with Functional Coatings and Their Possible Use in Distributed Sensor Technology †

**DOI:** 10.3390/s25237367

**Published:** 2025-12-03

**Authors:** Sandy Alomari, Kenny Hey Tow, Joao Pereira, Miguel Soriano-Amat, Tedros Weldehawariat, Korina Hartmann, Remco Nieuwland, Åsa Claesson

**Affiliations:** 1 RISE Research Institutes of Sweden, RISE Fiberlab, Fibervägen 2-6, 824 50 Hudiksvall, Sweden; kenny.heytow@ri.se (K.H.T.); joao.pereira@ri.se (J.P.); miguel.soriano.amat.rise@gmail.com (M.S.-A.); tedros.weldehawariat@ri.se (T.W.); asa.claesson@ri.se (Å.C.); 2 Department of Applied Physics, KTH, Roslagstullsbacken 21, 106 91 Stockholm, Sweden; 3 United Fiber Sensing B.V., Spui 37, 2511 BL The Hague, The Netherlands; korina.hartmann@unitedfibersensing.com (K.H.); remco.nieuwland@unitedfibersensing.com (R.N.)

**Keywords:** optical fiber, functional coating, distributed sensor, draw tower, hydrogen

## Abstract

Functional coatings on optical fibers enable selective detection of environmental and chemical parameters, but their use is typically limited to point or quasi-distributed sensing due to localized deposition techniques. In this work, we demonstrate a possible transition towards full-length functional coatings on optical fibers using a draw tower process, enabling their potential use in distributed sensor technology. An optical fiber with Pt:WO_3_ nanocomposite polymer functional coating is employed as a proof of concept. The results demonstrate the successful application of this functional coating along hundreds of meters of fibers using a draw tower. When integrated into a distributed sensing configuration, the Pt:WO_3_ fiber exhibited a clear change in response with varying hydrogen concentrations from 1% to 4% H_2_, with a temperature increase of 2.5 °C at 4 vol.% indicating a promising performance for distributed hydrogen leak detection. This approach opens new opportunities for applying other functional coatings over extended fiber lengths using draw towers, which could be exploited for novel distributed sensing applications.

## 1. Introduction

Standard coatings on optical fibers are mainly used to protect fibers against handling damage, mechanical stress, and environmental exposure and can sometimes serve as sensors for parameters like relative humidity [1] and temperature [2]. However, standard coatings are designed to be insensitive to changes in the ambient, and their use as active sensor materials is limited.

To overcome this limitation, modified coatings have been used to enhance the sensing performance of standard coatings [3]; functional coatings incorporating active materials such as metals [4], metal oxides [5], nanoparticles [6] or nanocomposite [7] have been developed for selective detection of specific physical, chemical or environmental parameters.

Functional coatings are commonly applied on fiber using conventional coating techniques such as dip coating [8], drop casting [9], sputtering [10], or sol–gel processing [11]. These techniques are typically not suitable for production of long lengths of functionally coated fibers but have successfully been used to manufacture point and quasi-distributed fiber sensors including fiber tips [12], fiber Bragg gratings (FBGs), Mach–Zehnder interferometers (MZIs), and long-period gratings (LPGs). Such sensors have been used for point detection of species such as methane and carbon dioxide (CO_2_) [13], ammonia [14], hydrogen [15], acetone vapor [16], and heavy metal ions [17,18].

Extending functional coatings to long-length fibers is essential for enabling fully distributed optical fiber sensing (DOFS) [19]. DOFS techniques allow continuous and real-time measurements along the entire length of the sensing fiber, which make them attractive in many industrial [20], environmental [21], safety [22], and other large-scale applications. Integrating functional coatings into such DOFS systems could significantly expand the capabilities of DOFS.

Using a fiber draw tower offers a promising route to achieve continuous long lengths of functionally coated optical fibers, suitable for DOFS. Such transfer, from short pieces of fiber to continuous production, requires careful process development, customized for each functional coating. This paper will discuss general considerations for this process development, highlighting possible limitations of both the coating process and the distributed sensing application.

Among various functional coatings, we identified platinum-doped tungsten trioxide (Pt:WO_3_) as a suitable candidate for a case study of the transfer from point sensor to DOFS. The Pt:WO_3_ nanocomposite exhibits an exothermic reaction in the presence of hydrogen gas, resulting in a localized temperature rise [23]. This temperature increase upon hydrogen exposure makes this coating particularly suitable for DOFS based on Raman Optical Time Domain Reflectometry (R-OTDR), which is highly sensitive to distributed temperature variations. Furthermore, previous studies have successfully dispersed Pt:WO_3_ nanoparticles in polyimide matrices [24], a standard coating material used in fiber draw towers, facilitating integration of this functional coating into the draw tower process.

## 2. Applying Functional Coatings for Distributed Sensing

Producing fibers with functional coatings using a draw tower requires optimization of several parameters to produce long fibers while maintaining mechanical integrity and sufficient optical performance.

**Figure 1:**
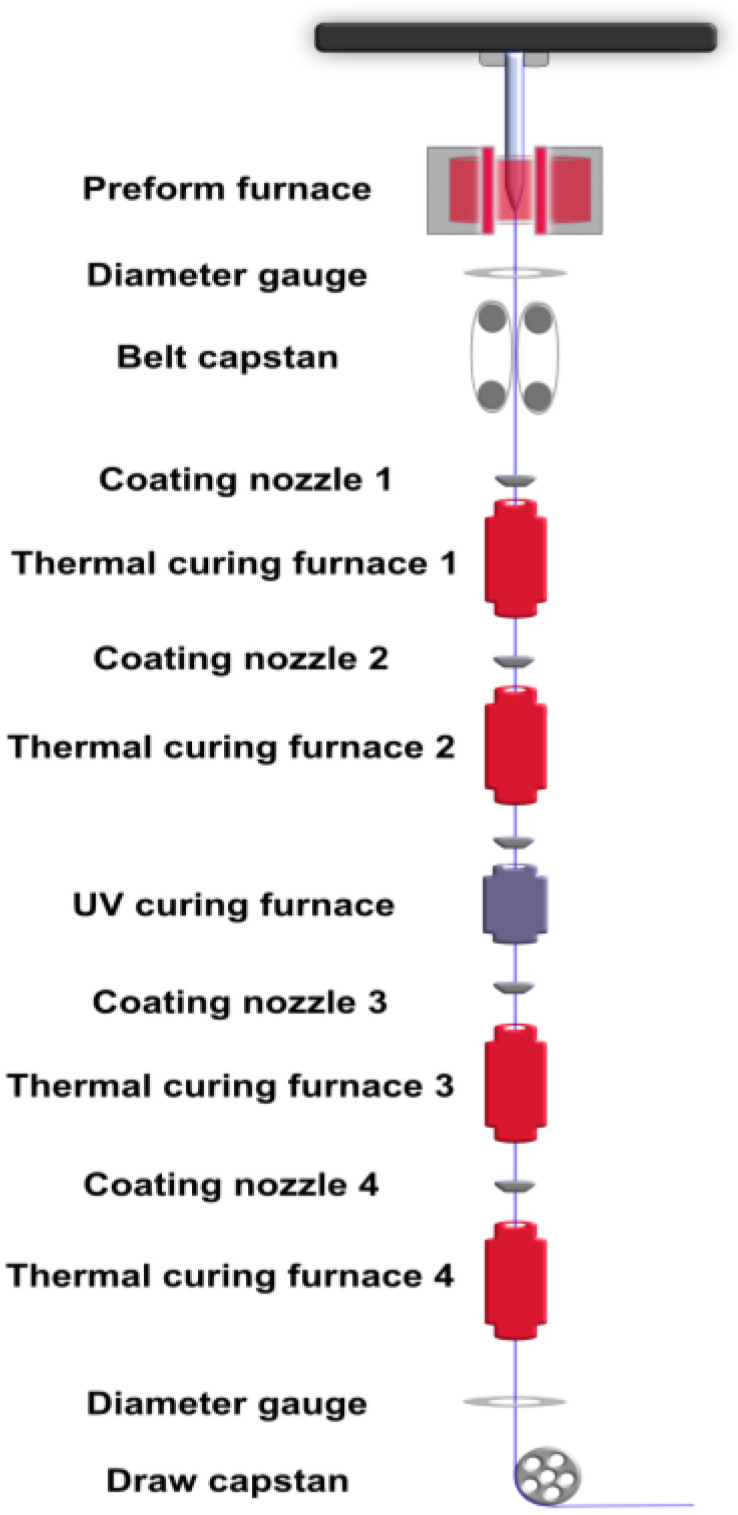
Schematic picture of one of the 17 m-tall optical fiber draw towers at RISE, used in this work, showing five coating nozzles that can be used to apply coating material on the fiber, four thermal furnaces, and one UV furnace to cure the coatings.

This section discusses quality parameters that are important to consider when using a full-size draw tower (Figure 1) to produce long lengths of mechanically robust, optically viable, functionally coated fibers suitable for DOFS. Each material system and sensor configuration will set specific requirements on the fiber parameters such as amount of functional material per meter fiber, optical loss tolerance of the system, and requirements on coating homogeneity. Although each coating system and each set of fiber parameters is unique, it is helpful to give some guidelines for the in-tower coating process. We restrict this discussion to polymer-based coating systems. The target is to manufacture fibers that are defect-free and robust, with good optical performance and adequate and reliable DOFS functionality.

General coating considerations: Coating thicknesses up to hundreds of micrometers with variations of ±1 to ±3 μm around the target thickness can be achieved in fiber draw towers. The necessary coating process will depend on many parameters, e.g., material properties (surface tension, viscosity), drying and curing behavior, coater nozzle type and size, and fiber draw speed [25]. For example, usable coating viscosities can range from very thin (e.g., 10 cP) to quite thick (e.g., 2500 cP) liquids. If required, fibers can be designed with several layers of coating. Draw speeds can range from less than 1 m/min to several hundreds of meters per minute or faster. Drying and curing of the coating material should be either thermal—or UV-based to be able to utilize equipment already installed in many specialty fiber draw towers. The drying/curing stage should achieve complete drying and/or polymerization without emitting hazardous gases or residues, ensuring a safe manufacturing environment. Adequate adhesion between the coating and fiber is also essential, and it is not uncommon to use adhesion promoters [25] in the coating.

Considerations specific to composite coatings: Functional coatings that rely on a dispersion or mix of materials must be homogeneously dispersed or fully dissolved in a chemically stable solution that is safe to use in a fiber draw tower. When using composite coatings with dispersed particles, particle size and the size distribution within the functional material can influence the uniformity of the coating and the efficiency of the sensor. Clustering or sedimentation of particles in the mixture during the draw process can lead to varying coating quality and a fiber with optical microbend losses [26] and unreliable sensor performance. Another important consideration, especially when using coatings with particles inside, is the risk of mechanically damaging the glass and limiting the durability of the fiber. To reduce this risk, one can first apply an inner protective layer with standard coating and then add the functional coatings on top, either in the same process or in an up-coating procedure where coatings are added to a ready-made fiber.

When designing a distributed optical fiber sensor, it is practical to base it on DOFS interrogation techniques for temperature and/or strain, where there are well-established commercial interrogators. The functional coating is then used to induce a measurable change in temperature or strain in the fiber at a specific measurand. The choice of interrogator is determined by the type and magnitude of the induced change. Rayleigh-based [27] and Brillouin-based DOFS systems [28] can be used to measure both temperature and strain changes in fiber with functional coating. Raman-based systems [29] are primarily temperature-sensitive and can be used to mitigate cross-sensitivity between strain and temperature. These DOFS systems are typically optimized for conventional low-loss telecommunications-type fibers. In contrast, a functionally coated fiber can display increased optical attenuation and slightly varying coating and optical properties along its length that can directly impact the performance of the system. Increased optical loss reduces the effective measurement range of the system, and varying optical and functional performance can lead to unreliable results.

Beside the operational range of the system, the magnitude of the transduced change in the optical parameters of the fiber with functional coating is also crucial. If the response is too small, the DOFS system will fail to distinguish small variations in the sensed parameter, and too much response can saturate the measurements. This can be optimized by adjusting coating thickness and the concentration of functional components inside the coating. Incorporating pores into the coating can further boost response magnitude by enlarging the analyte interaction surface area. The response must exceed the interrogator’s detection threshold, as a weak signal results in a low signal-to-noise ratio, degrades measurement precision, and reduces the sensor length. If the coating varies along the length of the fiber, the response may need to be averaged over a certain distance; this will lead to a lower spatial resolution and more signal averaging to compensate for such variation.

Other parameters, such as the response time of the DOFS instrument, can be important in certain applications. Together, these parameters define the reliability, sensitivity, accuracy, and repeatability achievable in the designed DOFS system.

In summary, integrating functional coatings into the fiber draw tower process enables production of long-length sensing fibers compatible with DOFS interrogation, thus extending the applications of such functional coatings.

## 3. Case Study: Pt:WO_3_ Polymer Coating

A nanocomposite Pt/WO_3_ polymer functional coating has been previously reported as a hydrogen sensor when deposited on fiber Bragg gratings (FBGs), with sensor responses measured over a hydrogen concentration range of 0.1 to 4 vol.% [30]. This coating was modified to be compatible with the draw tower process, applied using a draw tower, and then the resulting fiber was tested in a distributed sensing configuration.

### 3.1. The Preparation of the Functional Coating

**Figure 2:**
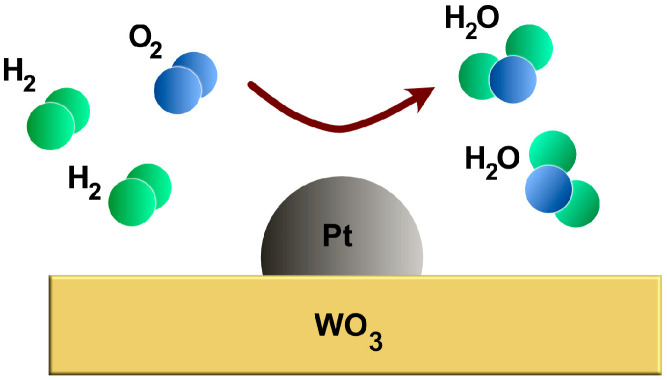
The exothermic catalytic reaction occurring on the surface of platinum (Pt) supported by tungsten trioxide (WO_3_) in the presence of H_2_, resulting in the release of water and a substantial amount of energy as heat. This temperature increase can be measured, enabling the calculation of hydrogen concentration in response to it.

In our experiments, Pt/WO_3_ dispersed in polyimide was used as the functional sensor active material to sense hydrogen gas. The sensing principle is straightforward, with hydrogen molecules first dissociating into hydrogen atoms by the platinum (Pt) catalyst. These hydrogen atoms then interact with tungsten trioxide (WO_3_), resulting in the formation of H_x_WO_3_ or WO_3−x_ [23]. This exothermic reaction (Figure 2) between Pt-loaded WO_3_ and surrounding hydrogen can be expressed as follows:

$${WO}_{3}+xH_{2}\overset{Pt}{\to }{WO}_{3-x}+xH_{2}O\;{WO}_{3-x}+\frac{x}{2}O_{2}\overset{Pt}{\to }{WO}_{3}$$

By measuring the variation in temperature caused by this exothermic reaction, hydrogen concentration can be calculated. Once the hydrogen is removed, the tungsten oxide oxidizes and WO_3_ is restored. Other gases like CO and CH_4_ do not interact significantly with Pt-WO_3_ at room temperature [31], which is promising for the selectivity of the functional chemistry, but this needs to be validated for these and other gases at the temperature range in which the sensors will work.

The nanocomposite functional coating was prepared following an approach similar to that described by Hartman [24]. Although the prepared mixture closely resembles the one Hartman used for point hydrogen sensing [24], some adjustments were made to the components to mitigate safety concerns in draw towers, particularly those that could lead to potential HF generation. In our experiments, platinum (Pt) was deposited on manually grinded tungsten oxide (WO_3_) powder. Hexachloroplatinic acid was mixed with the WO_3_ powder at a molar ratio of 1:10 and annealed at 400 °C for 1 h. The resulting Pt-loaded WO_3_ was then grinded and dispersed into polyimide (PI) precursor (40 wt% relative to WO_3_: Pt). The PI binds the Pt-WO_3_ to the fiber surface and its matrix controls the gas diffusion to the catalyst’s active sites. Polyethylene glycol (PEG) with a low molecular weight (Mw = 300 g/mol) was employed as a porogen to facilitate the formation of the porous structure. PEG decomposes during the curing process, leaving a network of pores within the PI matrix that increases the specific surface area of the material to provide more active sites in contact with hydrogen and improve the sensitivity of hydrogen response. The final coating mixture was inspected under a microscope to evaluate its homogeneity and overall quality and was used after that in the draw tower process.

### 3.2. The Application of the Functional Coating on Optical Fiber

The target was to deposit this functional coating on long lengths of optical fiber in a continuous process in a fiber draw tower, using equipment (coating nozzles, curing furnaces, gas flushing, etc.) already equipped in the tower, and to validate that the sensing functionality is maintained in a DOFS configuration. Given that the coating contains metal and metal oxide particles, which could potentially cause mechanical damage to the fiber, the fiber was designed with an inner protective layer of pure polyimide, protecting the glass from mechanical damage. The functional coating was then applied on top of this protective layer in an up-coating procedure.

Low-cost glass preforms were used in the initial process development to optimize the up-coating process. Once the process parameters were fine-tuned, the functional coating was applied in-tower on FBG, enabling evaluation of the coating’s performance as a point sensor in a simple and low-cost setup. After confirming a satisfactory response, multimode fibers were up-coated and used in distributed sensing trials using a R-OTDR configuration.

As an initial test of the coating functionality, a commercial polyimide FBG was spliced to a standard single-mode polyimide coated fiber (SMF-28 type) from both ends and then up-coated by feeding the fiber through the draw tower using nozzle 1 (Figure 1) and curing the coating in furnaces 1 and 2. The draw speed was set at 20 m/min, the nozzle diameter to 240 μm, and the thermal furnaces at 350 °C and 400 °C, respectively.

To prepare the DOFS fiber, a protective coating was first applied to the fiber with a three-layer structure consisting of neat polyimide coatings. The fiber fabrication was conducted at a preform furnace temperature of 2050 °C and a draw speed of 30 m/min. Polyimide layers were applied sequentially through nozzles 1, 2, and 3 (Figure 1), with nozzle diameters of 180, 190, and 200 μm, respectively, and all associated thermal furnaces maintained at 500 °C under nitrogen. This process produced a uniform fiber with three PI coating layers at a total of 156 μm in diameter.

The functional coating was then applied in an up-coating procedure using the same draw tower. Using nozzle size 240 μm and an up-coating speed of 20 m/min, and furnace temperatures of 350 °C and 400 °C under nitrogen, the resulting fiber had a total thickness in the range of 164 μm to 246 μm. Three batches were up-coated using the same parameters with an up-coated length of approximately 300 m per batch, constrained by the availability of the functional up-coating material. Each batch consumed approximately 200 g of the Pt/WO_3_ polyimide coating.

### 3.3. Studying the Coating Quality and the Optical Performance

The quality of the produced fiber was evaluated using optical microscopy and scanning electron microscopy (SEM) to assess surface uniformity, coating integrity, and the presence of any defects.

**Figure 3:**
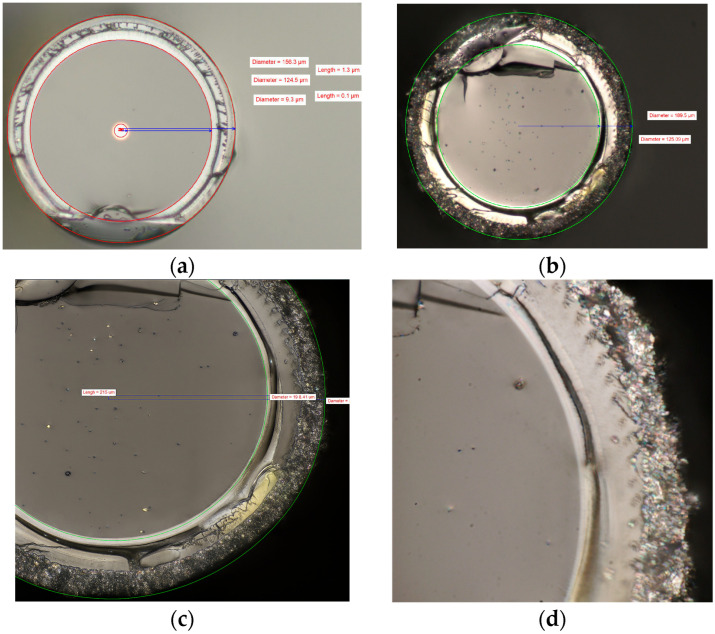
Cross-section images taken by optical microscopy of the fiber before (**a**) and after (**b**–**d**) up-coating with Pt/WO_3_ functional coating.

The cross-sections of the fiber before (Figure 3a) and after the up-coating process (Figure 3b–d) were studied in an optical microscope using 20×, 50×, and 100× magnification lenses. The images showed an almost radially concentric layer of nanocomposite coating surrounding the original polyimide coating. A short section of the fiber was then examined using SEM and EDS to assess the uniformity and microstructure of the coating surface.

**Figure 4:**
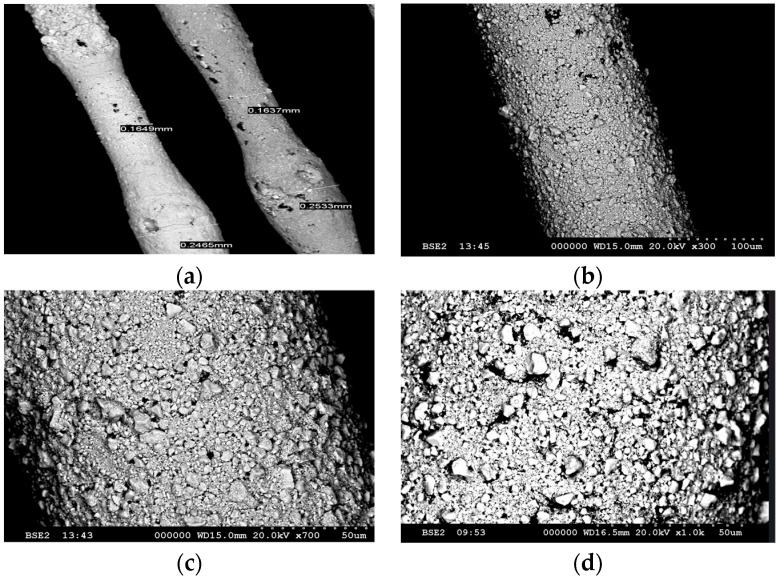
SEM images of the up-coated fiber with different magnifications. (**a**) The 60× magnification image showing variation in the coating thickness, sporadically appearing along the length of the up-coated fibers, an issue to be resolved by adapting coating viscosity and draw parameters. (**b**–**d**) Images taken at 300×, 700×, and 1000× magnification, respectively, illustrating a rough morphology of the coating surface as well as the variation in particle sizes on the surface of the functional layer.

The SEM images (Figure 4) demonstrate the variation in coating thickness and the particles sizes in the coating along the length of the fiber. In Figure 4a, the coating thickness varies from approximately 164 μm at the thinnest region to 246 μm at the thickest region of the up-coated fiber. Such drastic variations in coating thickness can sometimes be attributed to Plateau–Rayleigh instability in the coating [32]; in our case, we have the added complexity of the functional particles that introduces uncertainties in coating homogeneity and local variations in viscosity and surface tension. Further optimizing the coating mixing process, potential introduction of chemical modifiers, and careful optimization of nozzle size, coating viscosity, and draw speed is needed to mitigate the coating thickness variations. Figure 4c,d show the rough morphology on the surface, with particle sizes ranging from a few microns up to 10 μm. The morphology can potentially be made better by improving the grinding and sieving of the metal and metal oxide powder, by filtering the particles before mixture, and/or introducing chemistry to reduce particle clustering.

Since tungsten and platinum are the active components in the coatings, their distribution over a 3 cm up-coated sample was investigated using energy-dispersive spectroscopy (EDS). The molar ratio of Pt/WO_3_ in our coating was calculated and compared with the ratio in other publications [33].

**Figure 5:**
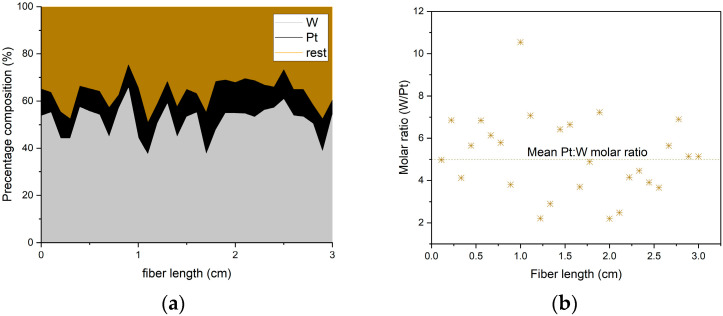
EDS results of a 3 cm segment of the up-coated fiber. (**a**) The weight percentages (wt%) of platinum and tungsten along the up-coated fiber plotted based on EDS results, revealing a variation in both elements’ concentrations along the 3 cm section. (**b**) The molar ratio between Pt and W calculated from the EDS results along a 3 cm section of the up-coated fiber, revealing a variation in the ratio from 1:2 to 1:10.

The Pt/W molar ratios were calculated from the Pt and W weight percentage and plotted along the 3 cm section (Figure 5); the Pt/W molar ratio varies widely from 1:2 to 1:10. We assume that on a macroscopic level, our fiber has a mean Pt:W molar ratio of approximately 1:5, consistent with the ratio reported to yield the highest sensitivity among the other tested ratios by Dai and his colleagues [33].

The optical properties of a standard 50/125 μm graded index multimode fiber up-coated with the functional coating were characterized, including attenuation loss, numerical aperture (NA), and mode field diameter (MFD) at 1550 nm. Attenuation loss was measured at 850 nm and 1300 nm using Optical Time-Domain Reflectometry (OTDR, Nettest CMA4000, GN NetTest, Utica, NY, USA), while NA and MFD were determined using an optical fiber analysis system (PK 2500, Photon Kinetics, Beaverton, OR, USA) and compared to a reference standard MM PI fiber (Table 1).

The NA and MFD values in Table 1 were not affected by up-coating the fiber with the functional coating; as expected, the variations are well within normal variations between preform batches and fiber draws. However, we observe a significantly higher attenuation loss for the up-coated fiber at both wavelengths, compared with the standard PI fiber. This higher attenuation can probably be attributed to micro-bending losses induced by the functional coating. Despite the increased attenuation losses, the functionally coated optical fibers fabricated for this study can still be probed by commercially available interrogators. The higher loss reduces the measurement range from over 30 km with the standard PI-coated fiber to approximately 1 km for the functionally coated optical fibers if we consider a dynamic range of 10 dB for the DOFS system and the loss of these experimental fibers. Despite this limitation, a 1 km measurement range is sufficient for applications such as monitoring hydrogen storage tanks or local distribution networks. Further optimization of the coating process such as employing smaller particle sizes and enhancing coating homogeneity are expected to reduce these losses and thus extend the effective measurement distance in the DOFS system.

**Figure 6:**
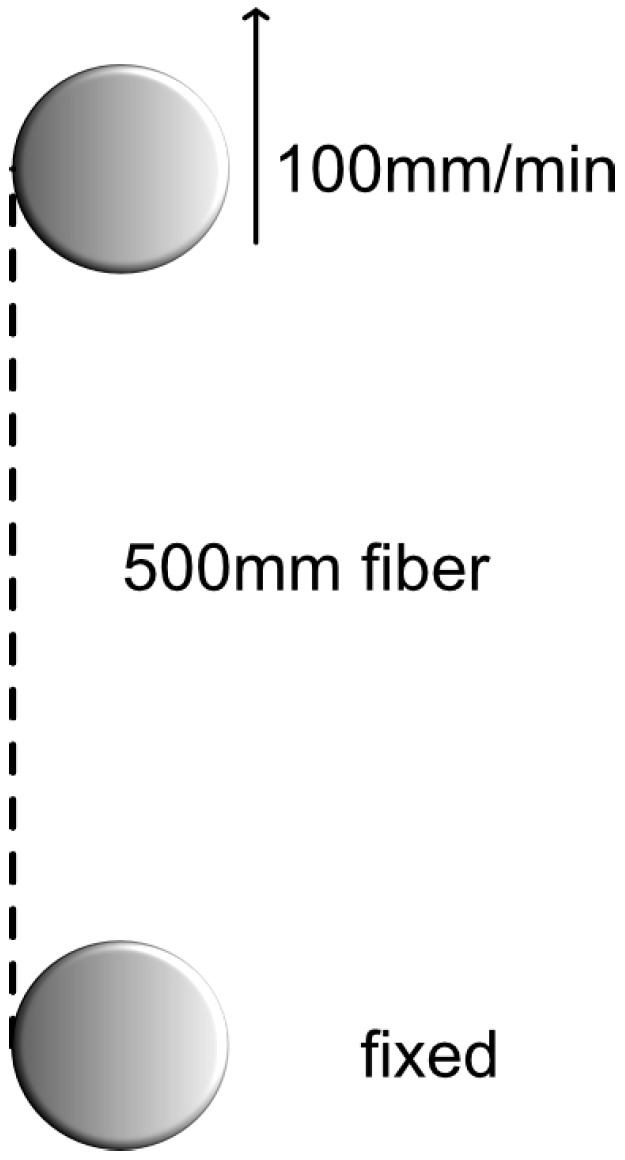
Schematic picture (left) of the tensile strength test setup with extension fixed at 100 mm/min.

The tensile strength of the reference PI and the up-coated fiber was measured using a standard setup at RISE (Figure 6). The setup consists of two wheels with 50 cm distance in between: one is fixed and the other one can move vertically while pulling the fiber with a fixed extension rate of 100 mm/min.

The results show an average tensile force of 68.8 N for the up-coated fiber compared to 68.7 N for the reference fiber, corresponding to a tensile strength of about 5.6 GPa for both fibers. This indicates that the up-coating process did not affect the mechanical properties of the fiber.

The results demonstrate that the Pt/WO_3_ functional coating can be successfully applied in-line during fiber up-coating without compromising the mechanical strength of the fiber. The optical NA and mode field parameters are not affected, but a significantly increased optical loss is observed, limiting the potential range of a DOFS based on these fibers. Future work will focus on enhancing coating uniformity along the fiber, refining particle size distribution, and optimizing the distribution of active elements within the coating.

### 3.4. The Validating of the Point Sensing Concept

To verify the compatibility of the functional coating with the draw tower up-coating process, its functionality was first tested on FBGs. The interaction between the functional coating and the hydrogen gas was investigated by testing the up-coated FBG in a controlled H_2_/air gas mixture. The FBG was flushed with dry air before being flushed with 5 vol.% hydrogen. The hydrogen flow was then stopped, and the system was allowed to cool down. The experiment was conducted at room temperature and atmospheric pressure and the FBG response was monitored using a commercial FBG interrogator.

**Figure 7:**
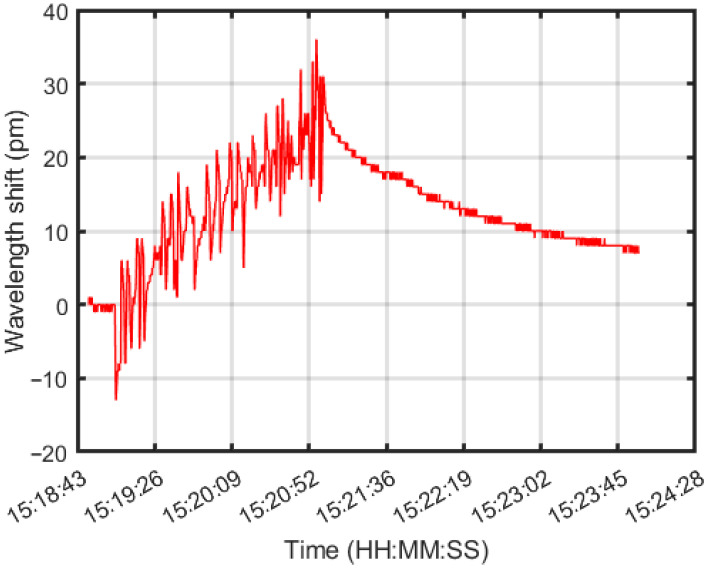
The up-coated FBG wavelength shift over time. The initial noise can be attributed to vibrations due to the flushing of H2/air mixture across the FBG. Exposure to 5 vol.% hydrogen gas shows an increase of 35 pm, which corresponds to an approximate temperature rise of 3.5 °C.

The wavelength changes in the up-coated FBG during the experiment, measured as a function of time (Figure 7), showed a wavelength increase of 35 pm in the presence of 5 vol.% hydrogen. Given that the typical sensitivity of an FBG at 1550 nm is around 10 pm/°C [34], the observed 35 pm wavelength corresponds to a temperature rise of about 3.5 °C on hydrogen exposure, indicating that the up-coated FBG can function as a hydrogen point sensor.

### 3.5. Towards Fully Distributed Optical Fiber Sensing Systems for Hydrogen Leak Detection

Since the functional materials coated on the optical fiber undergo an exothermic reaction upon exposure to hydrogen, causing a rise in the fiber temperature, R-OTDR system sensing was chosen as the DOFS system to evaluate our fiber.

As discussed above, the high attenuation loss, coating diameter variations, and microstructural variations in Pt:W ratio and concentration (illustrated in Figure 5) will give uncertainties to the absolute values in the measurements; however, a qualitative validation of the sensor functionality of the DOFS was prepared.

The R-OTDR system was used in a dual-ended configuration where the laser pulses were alternately launched into the fiber from both ends and backscattered Raman signals (both Stokes and anti-Stokes) were collected from both directions [35]. By acquiring measurements in both forward and backward directions along the same fiber section, the system can compensate for the increased attenuation losses along the coated fiber and the splice losses. For cases like absorption in the coating or fiber micro-bending, these losses can be wavelength-dependent and can be partially mitigated.

**Figure 8:**
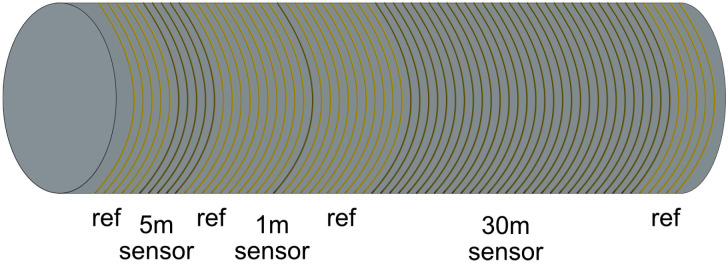
The FUT showing the three distinct up-coated lengths (5, 1, 30 m) spliced to equal lengths of standard MM polyimide fiber and wrapped around a plastic cylinder with an equal spacing of 1 mm between two windings.

A test fiber, fiber under test (FUT), was made by splicing three lengths (5, 1, 30 m) of the up-coated fiber with equal lengths of standard 50/125 MM polyimide fiber in between to create three distinct hotspots as shown in Figure 8.

The FUT was wrapped circumferentially around a polyvinyl chloride (PVC) plastic cylinder (Figure 8), with a spacing of 1 mm between two windings, before being placed in the test chamber.

**Figure 9:**
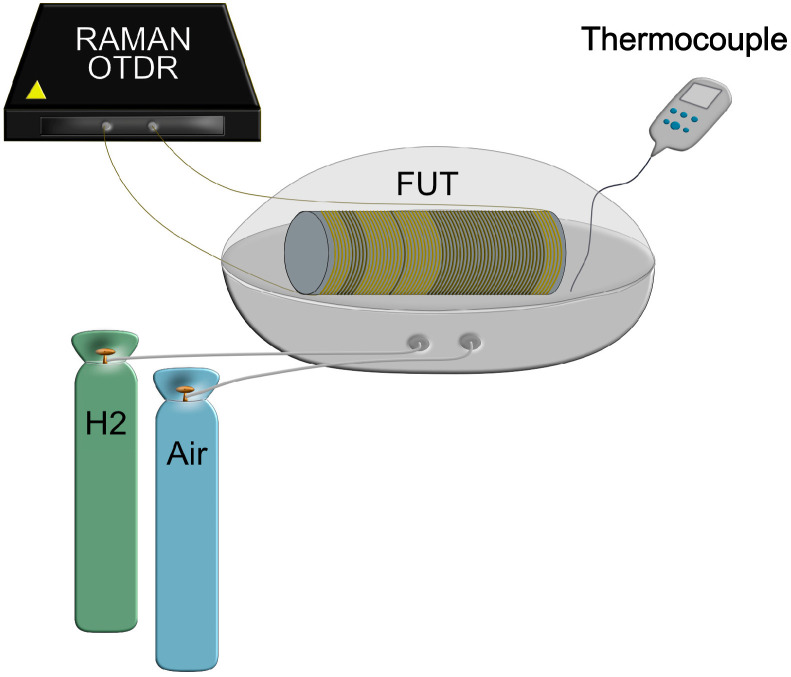
The experimental setup used to evaluate the up-coated fiber response in the presence of hydrogen includes an autoclave, a R-OTDR system, a thermocouple, and gas bottles.

The experimental scheme (Figure 9) consists of the FUT placed inside an autoclave in which hydrogen–air mixtures of different concentrations can be introduced at a controlled flow rate. To measure the distributed temperature along the FUT with improved temperature accuracy, the two ends of the FUT are connected in a dual-ended configuration to a commercial R-OTDR, which has a standard deviation of 0.1 °C under constant temperature conditions. A thermocouple with an accuracy of ±0.5 °C is also used to monitor the temperature inside the autoclave.

Oxygen analyzer (Servomex (2200/2223) oxygen analyzer, Servomex, Crowborough, UK) was used to measure the oxygen concentration during the test (O_2,meas_), and the hydrogen concentration was then calculated based on the known oxygen content in dry air (O_2,air_ = 20.95%) using the following equation.

$$H_{2}\%=100(1-\frac{O_{2,mesd}}{O_{2,air}})$$

Two distinct tests were performed using the FUT: the first test aims at evaluating the sensor’s response in the presence of hydrogen (H_2_), and the second investigates whether changing the hydrogen concentration will result in measurable variations in the FUT’s temperature response.

In the first test, the autoclave was initially purged to eliminate residual gases; then, a gas mixture consisting of air and 4 vol.% hydrogen was introduced and maintained for approximately 45 min before flushing the autoclave with pure air. During the test, the temperature along the FUT was recorded using the R-OTDR (N4586A, AP Sensing GmbH, Böblingen, Germany), the temperature inside the autoclave was monitored using a thermocouple, and the oxygen concentration was measured continuously.

**Figure 10:**
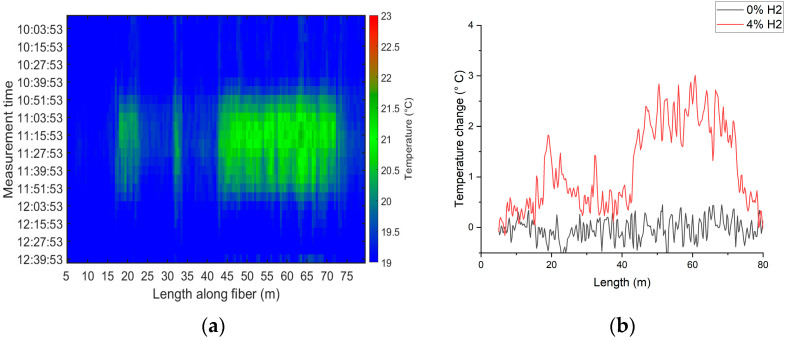
The measurements obtained from the first test were 4 vol.% hydrogen introduced in the autoclave for 45 min using R-OTDR. (**a**) The heatmap generated from plotting the measured temperature over time along the entire length of the FUT showing an increase in temperature in all three sections. (**b**) The calculated temperature changes at 0% and 4% H2 concentration along the FUT length indicating that the functional coating is responsive to the H2. Both Figure 10a,b were reprinted with permission from [36] © Optica Publishing Group.

The distributed temperature along the FUT was acquired every six minutes during the experiment time, with a spatial resolution of 0.5 m, and plotted in Figure 10a. The FUT heatmap demonstrates an increase in temperature over the up-coated sections at 4 vol.% hydrogen, compared to the reference PI sections. The heat map also indicates that the fiber cools down to its original temperature when the autoclave is flushed with pure air.

Figure 10b shows the temperature changes along the FUT, calculated by subtracting the distributed temperature along the fiber, measured between 10:00 and 10:15, from the corresponding temperature values measured at 4 vol.% hydrogen concentration (11:15) and after the fiber cooled down (12:30). The temperature change was higher along the 30-m coated hotspot, around 2.5 °C, as compared to the two other hotspot sections. The higher temperature response observed in the longer up-coated section can be attributed to its longer coating length, which likely leads to reduced heat dissipation and increased thermal accumulation compared to shorter sections. Additionally, the variations in temperature along the up-coated section can be explained by non-uniformities in coating thickness and composition along the fiber length. These spatial variations were confirmed by microscopic and SEM images, which showed differences in coating thickness, and by EDS results that illustrated fluctuations in the Pt/WO_3_ ratio.

For the second test, a gas mixture of air and hydrogen was introduced in the autoclave followed by gradually reducing the hydrogen concentration over time. Temperature measurements were acquired during the test using R-OTDR with an average reading every 0.25 m at three-minute intervals, and the hydrogen concentration was reported with time.

**Figure 11:**
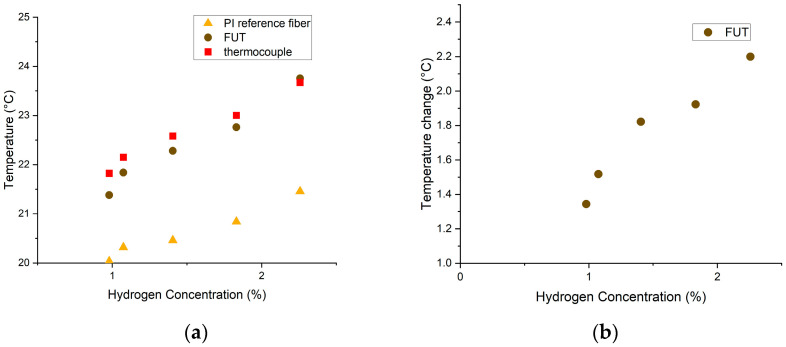
R-OTDR measurements obtained from the second test where the hydrogen gas concentration was gradually reduced. (**a**) shows the average temperature of the reference PI fiber, the FUT, and the thermocouple temperature, plotted as functions of the hydrogen concentration. In (**b**), the temperature difference between the FUT and the reference fiber is plotted as a function of the hydrogen concentration, showing a clear decrease in temperature as the hydrogen level decreases.

The average temperature measured in the central 10 m of the 30 m up-coated FUT section was plotted against the reported H_2_ concentrations and compared with the response of the PI reference fiber at the same H_2_ concentrations (Figure 11a). All sensors (the thermocouple and the two fibers) show a decrease in temperature with decreasing hydrogen concentration. This indicates that there is an overall temperature change in the autoclave during the experiment, likely from the heated FUT. To remove this uncertainty, Figure 11b plots the temperature difference between the FUT and the reference fiber vs. hydrogen concentration. From this graph, one can infer that the functional coating produces a distinguishable response for different hydrogen concentrations ranging between 1% and 2.5%, demonstrating its potential not only for detecting hydrogen but also to effectively monitor changes in hydrogen concentration within the systems where it is deployed.

While these results are not precise due to, e.g., the coating variations along the fiber length, they were qualitatively reliable and successfully reflected the expected response trend. Both tests’ results indicate the possibility of using such functional coating applied during a draw tower process for detecting H_2_ in a distributed system.

## 4. Discussion and Outlook

This paper describes the motivation for extending the use of functional coatings from limited length (e.g., FBG) to full-length fibers for distributed sensing. It discusses some of the challenges met when transferring functional coating processes from short manual processes to full-length draw tower fiber fabrication. We also discuss considerations when choosing the sensor instrumentation for measurable, reliable, and repeatable sensing response. Given the wide range of possible functional coatings, it is not feasible to define a single fiber draw process that suits every material. However, common challenges were addressed, including achieving coating homogeneity, ensuring reproducibility, maintaining good adhesion over extended fiber lengths, and preserving uniformity along the fiber length.

As a proof of concept, we describe the transformation of hydrogen leak detection technology from single-point FBG to fully distributed sensor probed by R-OTDR using a nanocomposite functional coating. We have presented qualitative results that indicate that the leak detection function is retained, with sensitivity variations along the fiber length. Achieving a more stable and higher response along the entire length of the sensor is crucial for ensuring the accuracy, detection ability, and reliability of precision sensing systems and is a key focus in future optimization efforts. Improving the coating preparation to ensure a better distribution of the active material and working on the coating process parameters to improve the uniformity and the coating quality over extended fiber lengths is an essential focus in future work. Cross-sensitivity towards other gases and the long-term reliability of the sensors will also be addressed.

Our case study showed the possibility of transferring a nanocomposite Pt:WO_3_ polymer functional coating from point to distributed sensor. A hydrogen concentration range of 1 to 4 vol.% was studied, with the functional coating producing an average temperature response of 2.5 °C at 4 vol.% hydrogen. Since the recommended alarm threshold for hydrogen detectors is 0.4% vol.% which represents 10% of the lower flammability limit, further studies should be conducted at such lower hydrogen concentrations. Additionally, more samples should be prepared and tested in the presence of hydrogen to verify the repeatability of the results. With the improvements mentioned above, the results could pave the way for a distributed hydrogen leak detection technology highly relevant in, e.g., large-scale industrial plants, hydrogen pipelines, and fuel storage facilities where early leak detection over extended lengths is critical.

The presented and demonstrated concept should be applicable to other functional coatings by choosing coatings capable of inducing measurable temperature, strain, or refractive index changes when interacting with a target analyte. Applying such functional coatings on optical fibers after tailoring them to meet the draw tower requirements will then enable fully distributed sensing capabilities, expanding the applicability of functional coatings to large-scale monitoring systems.

By tailoring the functional coating being used and optimizing the draw tower process for applying it on the optical fiber, it is possible to realize a new generation of distributed sensors for targeted detection based on the functional coating. Coupling these fibers with advanced signal interrogation methods such as Brillouin-, Rayleigh-, or Raman-based interrogators can extend the use of such functional coating to distributed sensing applications. Additional research will focus on expanding this material’s library and systematically studying its performance in long-length, field-deployable distributed fiber sensor networks.

In summary, this study establishes a foundation for the scalable fabrication of optic fibers coated with functional coatings, widely opening the field of distributed optical fiber sensing to new chemical and physical parameters.

## Figures and Tables

**Table 1:** Optical attenuation loss, numerical aperture (NA), and mode field diameter (MFD) of the up-coated 50/125 graded index MM fiber and a standard PI reference fiber of the same type, indicating that the application of the Pt/WO_3_ functional coating led to significantly increased optical signal attenuation.

	Loss at 850 nm (dB/km)	Loss at 1310 nm (dB/km)	Numerical Aperture (NA)	Mode Field Diameter (MFD)
Standard 50/125 μm MM PI	2.9	0.6	0.18	6.4
Up-coated 50/125 μm MM fiber	8.6	7.3	0.2	6.2

## Data Availability

The measurement data used in this study cannot be shared publicly because they contain proprietary information and are subject to confidentiality agreements with UFS. Access to the data may be granted upon reasonable request and with permission from the company.
